# Draft Genome Sequence of the Lignin-Degrading *Burkholderia* sp. Strain LIG30, Isolated from Wet Tropical Forest Soil

**DOI:** 10.1128/genomeA.00637-14

**Published:** 2014-06-19

**Authors:** Hannah L. Woo, Sagar Utturkar, Dawn Klingeman, Blake A. Simmons, Kristen M. DeAngelis, Steven D. Brown, Terry C. Hazen

**Affiliations:** a Department of Civil and Environmental Engineering, University of Tennessee, Knoxville, Tennessee, USA; b Graduate School of Genome Science and Technology, University of Tennessee, Knoxville, Tennessee, USA; c Biosciences Division, Oak Ridge National Laboratory, Oak Ridge, Tennessee, USA; d Microbial Communities Group, Deconstruction Division, Joint BioEnergy Institute, Emeryville, California, USA; e Biomass Science and Conversion Technology Department, Sandia National Laboratories, Livermore, California, USA; f Microbiology Department, University of Massachusetts Amherst, Amherst, Massachusetts, USA; g Department of Microbiology, University of Tennessee, Knoxville, Tennessee, USA; h Department of Earth and Planetary Sciences, University of Tennessee, Knoxville, Tennessee, USA

## Abstract

*Burkholderia* species are common soil *Betaproteobacteria* capable of degrading recalcitrant aromatic compounds and xenobiotics. *Burkholderia* sp. strain LIG30 was isolated from wet tropical forest soil and is capable of utilizing lignin as a sole carbon source. Here we report the draft genome sequence of *Burkholderia* sp. strain LIG30.

## GENOME ANNOUNCEMENT

*Burkholderia* species of *Betaproteobacteria* are ubiquitous in the environment and fulfill many different ecological niches. While some strains, like those of the *Burkholderia cepacia* complex, are pathogenic (1), other members of the genus have beneficial uses as biocontrol or bioremediation agents due to their wide array of secreted extracellular products or robust metabolic capabilities (2, 3). The strain presented here, *Burkholderia* sp. strain LIG30, was recently isolated from the protected Luqillo Experimental Rainforest soil in Puerto Rico using alkali lignin as a sole carbon source (4). Very few bacterial species are known to degrade lignin, and therefore this organism may contain novel lignin-degrading genes.

The genome was sequenced using the Illumina MiSeq platform, which generated 4,002,050 paired-end reads of 300-bp length. Quality-based trimming and genome assembly were performed using the CLC Genomics Workbench, version 7.0, to obtain 140 contigs, with an *N*_50_ contig size of 84,253 bp. Genes were identified using the Prodigal algorithm (5) as part of the Oak Ridge National Laboratory genome annotation pipeline. The predicted coding sequences were translated and used to search the NCBI nonredundant database, as well as the UniProt, TIGRFam, Pfam, PRIAM, KEGG, COG, and InterPro databases. These data sources were combined to assert a product description for each predicted protein. Noncoding genes and miscellaneous features were predicted using tRNAscan-SE (7), RNAMMer (8), Rfam (9), TMHMM (10), and signalP (11).

The draft genome is 5.5 Mb, with a 66.4% G+C content and 4,996 candidate protein-encoding gene models. Putative functions from COG functional groups were assigned to 73% of the candidate genes; 305 of the assigned genes were associated with secondary metabolite biosynthesis, transport, and catabolism. Based on EC numbers from PRIAM, 2 predicted genes encoded multicopper oxidases and 22 encoded putative peroxidases or catalases that may contribute to the lignin-degrading phenotype of *Burkholderia* sp. strain LIG30. The 16S rRNA gene, detected using RNAmmer 1.2 (12), has 99.41% identity to those from *B. ambifaria* strain AMMD and 99.34% identity to those from *B. cenocepacia* strain MC0-3 and *B. cenocepacia* strain HI2424 (13).

### Nucleotide sequence accession numbers.

This whole-genome shotgun project has been deposited at DDBJ/EMBL/GenBank under the accession no. JGVW00000000. The version described in this paper is version JGVW01000000.
